# Utilizing MV-FLOW^™^ and multidimensional ultrasound characteristics for prognosticating FET outcomes in RIF patients: Study Protocol for a cross-sectional study

**DOI:** 10.1371/journal.pone.0316028

**Published:** 2025-02-03

**Authors:** Ying Zhou, Li-Ying Liu, Hua-Ju Yang, Yuan-Yuan Lai, Di Gan, Jie Yang

**Affiliations:** 1 Sichuan Jinxin Xi’nan Women’s and Children’s Hospital, Chengdu, China; 2 Acupuncture and Tuina School, Chengdu University of Traditional Chinese Medicine, Chengdu, China; Teikyo University, School of Medicine, JAPAN

## Abstract

Recurrent implantation failure (RIF) is a common issue in frozen-thawed embryo transfer (FET). Prior to transfer, uterine endometrial receptivity of FET patients can be assessed using multimodal transvaginal ultrasound indicators to predict the success rate of the current FET cycle. Endometrial blood flow is a crucial element in evaluating endometrial receptivity. MV-FLOW^™^ is an advanced two-dimensional superb microvascular imaging technology that can detect and display blood flow in micro-vessels. The data for this study were obtained from an ongoing cross-sectional study comprising 323 RIF patients and 323 first implantation (FI) patients, who underwent transvaginal ultrasound before FET. We collected basic clinical data and multimodal ultrasound data from these patients as predictive features, with clinical pregnancy as the predictive label, for model training. Based on the above, this study aims to establish and validate a clinical prediction model for FET outcomes using support vector classification (SVC) algorithms, based on MV-FLOW^™^ and multidimensional transvaginal ultrasound imaging features. The objective is to determine the predictive role of multimodal transvaginal ultrasound in embryo transfer outcomes and provide evidence for the clinical application of MV-FLOW^™^.

**Trial registration:** *Trial Registration*: ChiCTR2400086401.

## Introduction

Recurrent implantation failure (RIF) marks a pivotal point in multiple embryo transfers (ETs), potentially impacting future success and requiring specialized diagnostics and treatments for live birth attainment [1]. Until recently, the definition of RIF has been multifaceted, contingent upon variables including female age, the nature of the transferred embryos, and whether the transfers were conducted with fresh or frozen embryos [2]. The heterogeneity of the biological basis for these definitions has led to challenges [3], affecting patients and reproductive medicine professionals, resulting in unreliable and contradictory outcomes and conclusions [1]. An initial study has revealed that, in the absence of discernible extrinsic factors, aneuploid blastocysts exhibit comparable rates of ongoing implantation and live birth following the first three consecutive single embryo ET. Among 4,429 women, this cohort demonstrated a cumulative implantation probability of 95.2% and a cumulative live birth probability of 92.6% [4]. These findings corroborate mathematical models derived from literature-reported implantation and live birth rates of aneuploid blastocysts [1]. Consequently, these studies suggest that the prevalence of "true" unexplained RIF within the IVF population is less than 5%, given that no statistically significant progressive decline in implantation or live birth rates was detected across the first three consecutive groups of aneuploid blastocyst transfers.

Nonetheless, certain scholars harbor reservations about this viewpoint [5] given that the study’s subjects all underwent costly preimplantation genetic testing for aneuploidies (PGT-A) and endometrial receptivity analysis (ERA) diagnostics, limiting their applicability across all in vitro fertilization (IVF) patients. In 2023, the European Society of Human Reproduction and Embryology (ESHRE) [6] recommended a 60% threshold for cumulative predicted implantation opportunity in recurrent implantation failure (RIF) to differentiate between euploid and aneuploid embryo transfers. Pre-transfer multimodal vaginal ultrasound indicators are routinely used in clinical practice to assess endometrial receptivity in FET patients, serving as a diagnostic tool to predict FET success rates. Endometrial blood flow, primarily assessed via 2D ultrasound following the Applebaum criteria [7], is crucial for evaluating blood flow velocity and direction through the Doppler effect. Endometrial blood flow is a crucial aspect of endometrial receptivity assessment, primarily through 2D ultrasound mode, following the Applebaum criteria for detecting and classifying endometrial blood flow signals. Its principle involves utilizing the Doppler effect of ultrasound waves to evaluate blood flow velocity and direction. Specifically, when the ultrasound beam encounters moving red blood cells, it generates a frequency change proportional to the blood flow velocity. By analyzing this frequency change, the blood flow velocity can be calculated, thereby assessing the richness of blood flow and vascular resistance. MV-FLOW^™^ is an advanced spatiotemporal coherent superb microvascular imaging technology capable of detecting and displaying blood flow in small vessels. Studies have shown [8–10] that MV-FLOW^™^ can display low-speed blood flow in normal or diseased tissues (such as tumors), with high sensitivity and resolution. Our previous research [11] observed the effects of acupuncture on MV-FLOW^™^ in RIF patients on the day before implantation, suggesting that MV-FLOW^™^ is a sensitive indicator of endometrial blood flow and that acupuncture can exert immediate regulatory effects on this indicator. However, the predictive value and advantages of MV-FLOW^™^ for embryo transfer remain unclear.

This study, grounded in a current cross-sectional investigation employing support vector classification (SVC) algorithms, aims to explore: (1) the predictive precision of MV-FLOW^™^ endometrial vascular distribution indices and/or endometrial vascular indices for FET outcomes in RIF patients; (2) the potential of these indices to categorize patients as either successful first implantation (FI) or RIF.

## Methods and materials

### Study design

**Fig 1:**
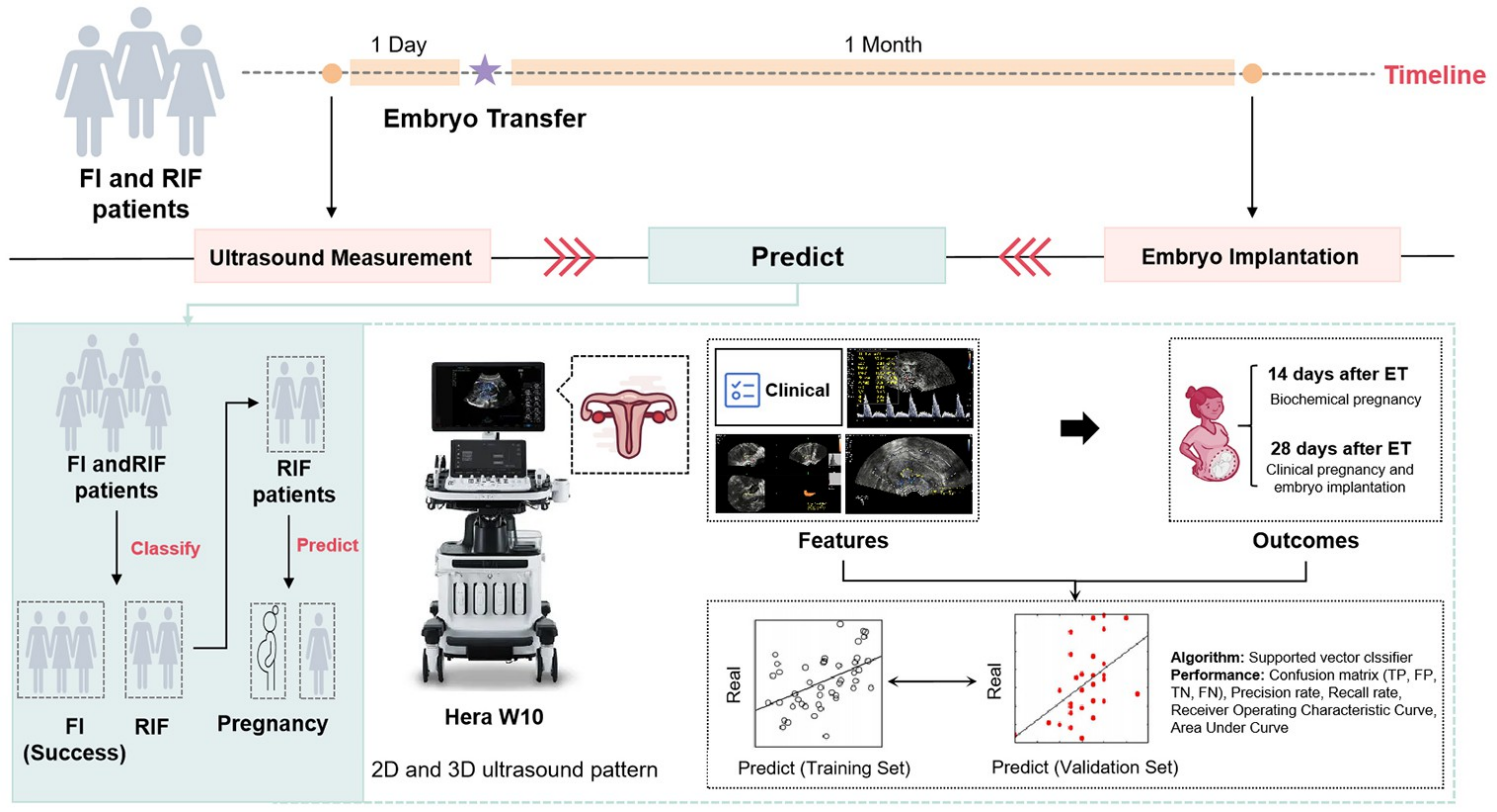
Study flow chart. Note: FI, first implantation; RIF, Recurrent implantation failure; ET, embryo transfer.

**Fig 2:**
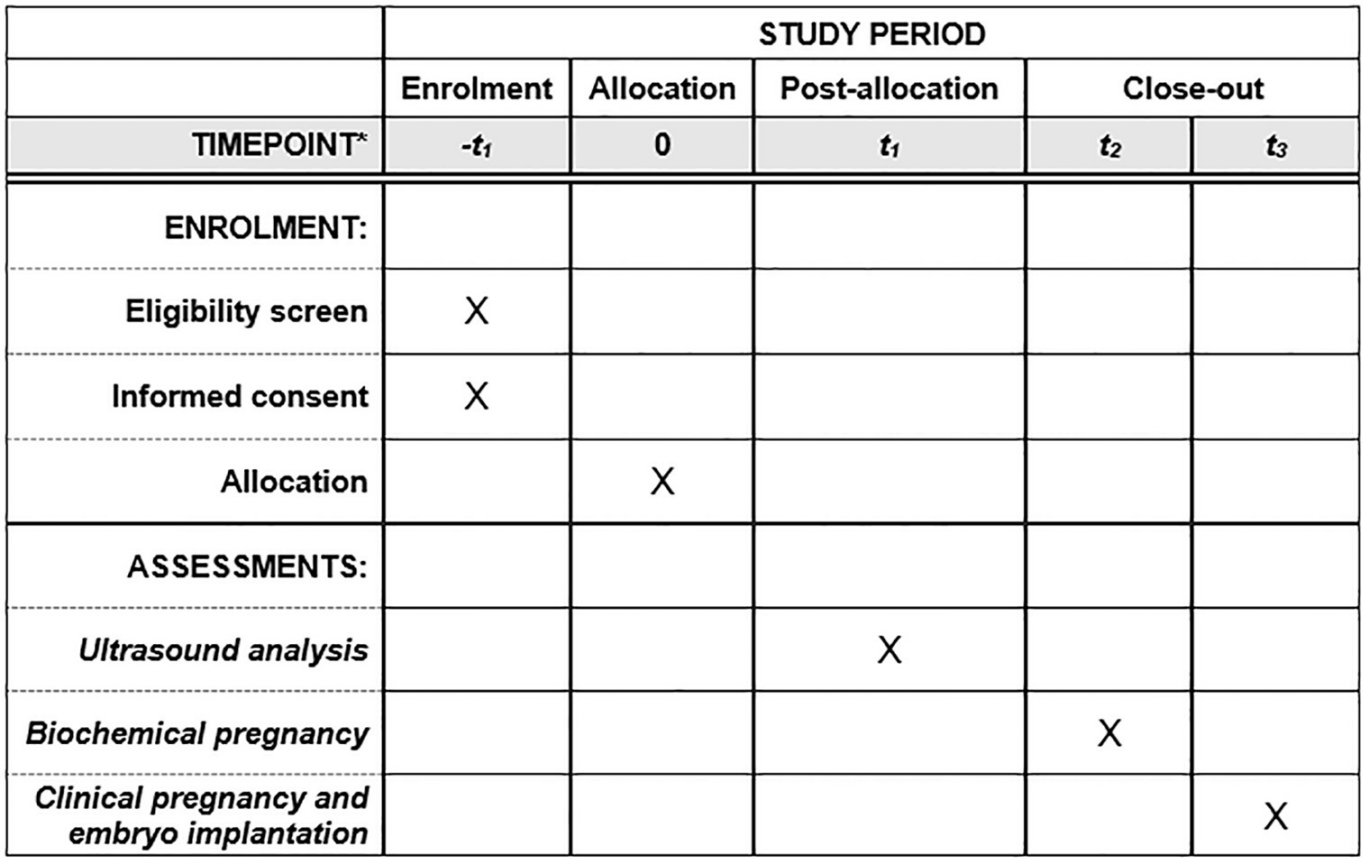
The schedule of enrolment, interventions, and assessments. Note: *-t1: Eligibility screen and informed consent before frozen embryo transfer; 0: Allocation at the beginning of frozen embryo; t1: Ultrasound analysis at the day before embryo transfer; t2: Biochemical pregnancy at 14 days following embryo transfer; t3: Clinical pregnancy and embryo implantation at 28 days after embryo transfer.

We will recruit a cross-sectional observational cohort of FI and RIF patients from Jinxin Sinan Women’s and Children’s Hospital in Sichuan who underwent FET. These patients will undergo transvaginal ultrasound examination, including assessment of endometrial MV-FLOW^™^ prior to embryo transfer in FET. First, the foundational clinical data of RIF patients, along with endometrial ultrasound-related indices, will serve as features to forecast the clinical outcome of FET. Subsequently, a clinical prediction model grounded in multidimensional ultrasound features will be developed. The efficacy of this model will be rigorously assessed and subjected to cross validation. Then, the characteristics observed under transvaginal ultrasound examination in successful FI and RIF patients will be employed as predictive indicators to ascertain whether they can effectively categorize successful FI and RIF patients post-transplantation. The dates of the recruitment are July 15^th^, 2024 to July 15^th^, 2025. We will also establish a flexible recruitment timeline, allowing for adjustments as needed, and regularly monitor and evaluate the recruitment progress, enabling us to identify and address any issues promptly. All obtained private data will be subject to the supervision of the ethics committee, and the study will be conducted after patients have signed the informed consent form. This trial was reviewed by the ethics committee of Sichuan Jinxin Xi’nan Women’s and Children’s Hospital (2023–006), and registered in Chinese Clinical Trial Registry (ChiCTR2400086401) (See S1 Table). Study flow chart see Figs 1 and 2 and S2 Table (the SPIRIT schedule of enrollment).

### Participants and sample size

#### Diagnostic criteria for RIF patients

According to the definition of RIF proposed by EHSRE 2023 [12], the transfer of embryos considered viable fails to result in a positive pregnancy test for a given patient and is insufficient to consider further investigation and/or intervention. Considering that the recommended threshold for the cumulative predicted chance of implantation for RIF is 60%, when a couple does not successfully implant by transferring a certain number of embryos and the cumulative predicted chance of implantation associated with that number is greater than 60%, then they should be advised to undergo further investigations and/or treatment options.

For embryos of unknown euploidy [13], RIF is diagnosed in female patients under 35 years old after three or more embryo transfers (ET); for those aged 35 to 39, after four or more ETs; and for those aged 40 and above, after six or more ETs. For embryos with known ploidy status [14], RIF is diagnosed after two or more ETs.

#### Diagnostic criteria for FI patients

FI patients are defined as patients who have not previously undergone fresh or frozen embryo transfer.

#### Inclusion criteria

Patients met all four following criteria will be included.

- Aged between 21 to 45 years who are undergoing FET;
- Diagnosed as FI or RIF patients;
- Using the hormone replacement treatment (HRT) or a GnRH-a down-regulating hormone replacement protocol for FET endometrial preparation;
- Signing an informed consent form.

#### Exclusion criteria

Patients met any one of the following criteria will be excluded.

- Diagnosed as severe primary or secondary conditions in the active phase, or mental disorders;
- Diagnosed with significant, untreated uterine malformations impacting uterine morphology, uterine fibroids exerting pressure on the uterine cavity, or ovarian cysts, as delineated by ultrasound;
- History of malignant neoplasms in any anatomical location;
- Presence of chromosomal abnormalities (excluding chromosomal polymorphisms).

#### Sample size calculation

This study is a cross-sectional trial to establish a predictive model for endometrial receptivity and pregnancy outcomes using SVC to predict clinical pregnancy. The ultrasound assessments of uterine arteries and endometrial thickness are crucial for pre-implantation evaluation of endometrial receptivity, a study involving 78 FET patients (resulting in 45 clinical pregnancies, 57.7%) utilized elastic ultrasound imaging and nine predictive indicators based on basic information, achieving a predictive accuracy of 76.92% [15]. Sample size estimation was conducted using “pmsampsize” in R software [16]. According to the definition of RIF, at least two failed ETs were diagnosed as RIF. Assuming a clinical pregnancy rate of 30%, a minimum of 323 RIF patients were required for sample estimation. In a 1:1 ratio, 323 FI patients were also included. The code for sample size calculation is referenced in S1 Fig.

### Vaginal ultrasound data acquisition

#### Instrument and collection preparation

The SAMSUNG Hera-W10 Doppler Ultrasound Diagnostic System, equipped with an EV3-10B vaginal probe operating at 3.3 MHz, will serve as the instrument for ultrasound data acquisition. Patients will be directed to empty their bladders and adopt the lithotomy position. Subsequently, a disposable condom will be placed over the ultrasound probe, which will be evenly coated with ultrasound gel. The probe will then be gently and slowly inserted into the subject’s vagina, aiming to reach the posterior fornix for scanning and measurements. This vaginal ultrasound examination will be performed by a seasoned gynecological ultrasonographer with over ten years of experience.

#### Ultrasound acquisition patterns and content

*2D ultrasound pattern*. In the two-dimensional grayscale mode, measurements of endometrial thickness, endometrial morphology, and endometrial motion will be conducted. Endometrial thickness will be determined by averaging three measurements of the distance between the anterior and posterior myometrium-endometrium interfaces, taken 1 cm from the endometrial apex. Detailed observation of endometrial echogenicity will be conducted, and the endometrium will be categorized into types A, B, and C based on the Gonen criteria [17]. Endometrial wave motion will be observed and documented for three minutes on the longitudinal midline section of the uterus with the probe stabilized and the patient breathing normally. Endometrial motion is classified into positive wave, negative wave, quiescent wave, bidirectional wave, local peristaltic wave according to the Ljland criteria [18].

In the two-dimensional color mode, with the uterus longitudinally bisected along its midline axis, the subendometrial region is demarcated as extending 3mm beyond the endometrial margin. Utilizing two-dimensional color Doppler flow imaging (CDFI), the perfusion of the endometrium and subendometrial areas will be assessed, classifying the flow patterns into Type I, II, and III according to the Applebaum criteria [7]. Subsequently, the CDFI is employed to delineate the uterine sagittal section, orthogonal to the long axis of the uterine cavity, where blood flow measurements are conducted. The color imaging technique is used to visualize the uterine arteries, with the sampling gate sequentially positioned at the cervix, both sides of the corpus, and the junction of the myometrium and endometrium to obtain stable blood flow spectrograms. Measurements are taken only after a minimum of consecutive stable waveforms are observed. The resultant parameters of uterine arterial blood flow encompass the peak systolic velocity (PSV), end diastolic velocity (EDV), time-averaged maximum velocity (TAMV), time-averaged peak velocity (TAPV), mean pressure gradient (PGmean), systolic/diastolic ratio (S/D), resistivity index (RI), and pulsatility index (PI).

The MV-FlOW^™^ technology employs advanced clutter filters to evaluate low-velocity blood flow signals. To facilitate MV-FlOW^™^ sample acquisition, the sensor is positioned and the imaging adjusted to focus on the endometrium and the uterus, thereby enabling a quantitative assessment of blood perfusion. The Volumetric Index of Microvascularity (VI^MV^) is computed automatically as the ratio of blood-filled pixels to the total number of pixels within the region of Interest (ROI; VI^MV^ = n_blood/n_total). By activating MV-FlOW^™^, the operator achieves optimal visualization of the vascular tree. In this study, the ROI encompasses the entire uterus, and the VI^MV^ is determined by the proportion of endometrial blood flow pixels to the total blood flow pixels within the uterus. The parameters for the MV-Flow on the Hera-W10 system are standardized as follows: Gain at 54; Frame Average set to 8; Output Power at 90; Dynamic Range of 50; Alpha Blending at 70%; Color Map selected as 2; LumiFlow set to 2; Mechanical Index (MI) at 1.3; Thermal Index (TIs) at 0.3; and Depth calibrated to 6 cm.

*3D ultrasound pattern*. Initiating the power Doppler flow imaging mode, the sensitivity is adjusted to a heightened state to observe the branching patterns of endometrial blood flow. Subsequently, the CrystalVue^™^ mode is activated to delineate the endometrial contour, yielding a three-dimensional representation of the uterine endometrium. This process automatically calculates the endometrial volume (EV), the vascularization index (VI), the flow index (FI), and the vascular flow index (VFI).

### Clinical data acquisition

We will systematically compily comprehensive clinical data for RIF or FI patients, encompassing their foundational details such as chief complaints, present illness history, past medical history, family history, allergy history, pertinent examination findings, comorbid conditions, and medication use. Additionally, we will meticulously document their prior clinical information from assisted reproductive technology (ART) treatments, including treatment protocols, cycle specifics, and outcomes.

### Outcome acquisition

We will obtain clinical outcomes, which include clinical pregnancy and embryo implantation at 28 days, as well as biochemical pregnancy at 14 days following embryo transfer.

### Data analysis

#### Data statistics

Data analysis was conducted using the SAS 9.4 statistical software (SAS Institute, Cary, NC, USA) by an independent statistician. Comparisons between the two groups will be performed as follows. For quantitative data, independent sample t-tests, analysis of variance, or Kruskal-Wallis H tests were employed; for categorical data, chi-square tests or Fisher’s exact probability method were utilized. All statistical analyses are two-sided, with *P* < 0.05 considered statistically significant.

#### Clinical prediction model

*Machine Learning Algorithm*. In this study, the SVC algorithm was selected for machine learning applications. All computations were executed using the Sklearn package within Python 3.10.

*Data Preprocessing*. Altogether four steps of data preprocessing were applied in this study. First, we will remove the outliers and organize the data. As for missing value filling, we will conduct KNN Imputer for continuous variables, and Simple Imputer for categorical variables. The Spearman correlation analysis was first conducted to evaluate the relationship between all predictive feature variables and clinical pregnancy outcomes (Spearman’s correlation coefficient more than 0.2 were significant). Eventually, we removed low-variance (Variance Threshold = 0.8) variables and highly correlated variables (r^2^>0.8).

*Prediction Variable Selection*. Thereafter, a SVC model was constructed using the remaining feature variables. The filtered feature subset was then input into the established model to rank the feature weights. These weights indicate the contribution of each feature to the determination of the SVC hyperplane. In accordance with the LIBSVM manual, SVC_coef was employed as the feature weight to compute the permutation importance of the features, with the feature exhibiting the maximum value of the mean ± standard deviation being deemed the most valuable predictive indicator.

*Model performance evaluation*. The dataset for the predictive model was partitioned into a training set and an internal validation set in a 7:3 ratio, with internal validation of the model conducted using cross-validation techniques. The accuracy of the classification results was assessed through the use of a confusion matrix. For binary classification tasks, sample cases can be categorized into four scenarios based on their actual class and the predicted class by the trained model: true positives (TP), false positives (FP), true negatives (TN), and false negatives (FN). The model’s accuracy was evaluated using the precision ratio (P) and recall rate (R). Ultimately, the F1 score was employed to synthesize both precision and recall. Additionally, the model’s performance was evaluated using the receiver operating characteristic (ROC) curve and the area under the curve (AUC).

## Discussion

Angiogenesis plays a pivotal role in the growth of the endometrium and embryo implantation [19,20]. During the transition of the endometrium from the proliferative to the secretory phase, there is a gradual increase in endometrial blood flow, and abundant endometrial blood supply is crucial for successful embryo implantation [21]. Dynamic transvaginal ultrasound monitoring of changes in uterine arteries, endometrium, and sub-endometrial angiogenesis can effectively assess endometrial blood supply. A meta-analysis [22] revealed statistically significant differences in endometrial VI, FI, VFI on the day of embryo transfer, and sub-endometrial vascular FI on the day of human chorionic gonadotropin (hCG) injection between pregnant and non-pregnant women following assisted reproductive technology treatment, suggesting a potential relationship between these blood flow parameters and pregnancy occurrence. However, some studies have questioned the relationship between endometrial blood flow indices under ultrasound and endometrial receptivity. For instance, Mayer et al. [23] found no statistically significant differences in endometrial and sub-endometrial VI, VFI, and FI on the day of transfer between pregnant and non-pregnant women in frozen embryo transfer cycles. Similarly, Zhang et al. [24] reported no statistically significant differences in uterine PI, RI, and endometrial and sub-endometrial VI, FI, VFI on the day of hCG injection between pregnant and non-pregnant groups, as well as between miscarriage and ongoing pregnancy groups, suggesting that blood flow-related parameters of the endometrium and sub-endometrium cannot accurately assess endometrial receptivity.

However, most prior studies have relied on retrospective analysis, potentially yielding biased results. Hence, prospective research remains essential to evaluate the feasibility and precision of ultrasound-derived uterine blood flow parameters in predicting the optimal embryo transfer timing in IVF. This protocol outlines a prospective, cross-sectional study that explores the role of ultrasound in pre-embryo transfer diagnostics. Building on this, we have pioneered the use of MV-FLOW^™^ to detect microvascular distribution within the endometrium and sub-endometrium, and validated its feasibility in assessing endometrial vasculature [11]. SVC [25] is a common supervised machine learning algorithm that seeks an optimal hyperplane in feature space to discriminate between different classes, characterized by excellent generalization capabilities. We propose to input all ultrasound-derived uterine blood flow parameters as flow features to predict embryo implantation outcomes and rates, thereby identifying the most predictive ultrasound indices for embryo implantation and discussing the diagnostic value of MV-FLOW^™^ in assessing endometrial receptivity. RIF is predominantly influenced by embryonic or uterine factors, with studies indicating abnormal blood flow parameters [26]. Our study extends this by investigating the clinical utility of these parameters in differentiating between successful first-time transfers and RIF cases, offering predictive markers for RIF identification.

In conclusion, while we have discussed the potential effects of MV-FLOW^™^ in predicting frozen embryo transfer outcomes in patients with recurrent implantation failure, it is important to consider the broader clinical implications should our study’s predictions prove accurate. Successful prediction models could revolutionize personalized treatment plans, allowing clinicians to tailor interventions more effectively and improve overall success rates in assisted reproductive technologies. This could lead to a paradigm shift in how recurrent implantation failure is managed, ultimately enhancing patient outcomes and satisfaction. Furthermore, the integration of MV-FLOW^™^ with multidimensional ultrasound features could pave the way for more comprehensive diagnostic tools, offering deeper insights into the underlying causes of implantation failure and facilitating more targeted therapeutic strategies.

## Supporting information

S1 Table Clinical trial registry (ChiCTR2400086401). (PDF)

S2 Table The table of SPIRIT checklist. (DOC)

S1 Fig The code for sample size calculation. (PNG)
