# Supplementary material for: Utilizing MV-FLOW™ and multidimensional ultrasound characteristics for prognosticating FET outcomes in RIF patients: Study Protocol for a cross-sectional study
Source: PLoS One. 2025 Feb 3;20(2):e0316028. doi: 10.1371/journal.pone.0316028 (PMC11790133; doi:10.1371/journal.pone.0316028)
Supplement: S1 Table — (PDF) [file pone.0316028.s001.pdf]

修改项目信息edit project

审核状态: 该项目通过审核
Project audit state: This trial is Successful.

* 填写语言: Language:	中文和英文/Chinese And English
* 注册号状态: Registration Status:	预注册/Prospective registration
* 注册题目:	基于MV-FLOW及多维阴道超声影像特征对FET患者冻融胚胎移植结果的临床预测模型构建及验证
* Public title:	Construction and validation of a clinical prediction model based on MV-FLOW and multidimensional vaginal ultrasound imaging fe
注册题目简写:	
English Acronym:	
* 研究课题的正式科学名称:	基于 MV-Flow对反复移植失败患者子宫内膜容受性的预测模型构建
* Scientific title:	Predictive modeling of endometrial tolerance in patients with repeated graft failure based on MV-Flow
研究课题的正式科学名称简写:	
Scientific title acronym:	
研究课题代号(代码): Study subject ID:	
在二级注册机构或其它机构的注册号: The registration number of the Partner Registry or other register:	
* 申请注册联系人:	刘璨莹
* Applicant:	Liu Liying
* 申请注册联系人电话: Applicant's telephone:	+86 186 2811 5041<br>请输入国际区号如:+86 xxx,区号与号码之间添加一个空格<br>Please enter the phone area code such as +86 xxx and add a space between the area code and the number
申请注册联系人传真:	
* 申请注册联系人电子邮件: Applicant's E-mail:	767000032@qq.com
申请单位网址(自愿提供): Applicant's website(voluntary supply):	
* 申请注册联系人通讯地址:	四川省成都市金牛区十二桥路37号
* Applicant's address:	37 shierqiao road, Jinniu District, Chengdu, Sichuan
申请注册联系人邮政编码: Applicant's postcode:	
* 申请人所在单位:	成都中医药大学
* Affiliation of the Registrant:	Chengdu University of TCM
* 是否获伦理委员会批准: Approved by ethic committee:	○ 是/Yes ○ 否/No
* 伦理委员会批件文号: Approved No. of ethic committee:	2023-006
* 批准本研究的伦理委员会名称:	四川锦欣西囡妇女儿童医院医学伦理委员会
* Name of the ethic committee:	Medical Ethics Committee of Sichuan Jinxin Sinan Women's and Children's Hospital

* 伦理委员会批准日期: Date of approved by ethic committee:	2023-02-06
* 伦理委员会联系人:	刘敬
* Contact Name of the ethic committee:	Liu jing
* 伦理委员会联系地址:	四川省成都市锦江区毕昇路66号
* Contact Address of the ethic committee:	66 Bisheng Road, Jinjiang District, Chengdu, Sichuan
* 伦理委员会联系人电话: Contact phone of the ethic committee:	+86 28 6550 6841<br>请输入国际区号如:+86 xxx,区号与号码之间添加一个空格<br>Please enter the phone area code such as +86 xxx and add a space between the area code and the number
国家FDA批准文号: Approved No. of SFDA:	(选填Optional)
国家FDA批准日期: Date of approved by SFDA:	选择日期 (选填Optional)
* 研究计划书: Study plan file:	已上传附件/Attachment（仅用于审核，不公开 for prejudication of registration only, will not be published）<br>上传与伦理审查对应版本的最新研究计划书
* 知情同意书: I informed consent file:	已上传附件/Attachment（仅用于审核，不公开 for prejudication of registration only, will not be published）<br>上传与伦理审查对应版本的最新知情同意书，如免除知情同意，请上传对应的免除知情同意申请
* 研究实施负责(组长)单位:	四川锦欣西囡妇女儿童医院
* Primary sponsor	Sichuan Jinxin Sinan Women's and Children's Hospital
* 研究实施负责(组长)单位地址:	成都市锦江区毕昇路66号
* Primary sponsor's address:	66 Bisheng Road, Jinjiang District, Chengdu, Sichuan
* 试验主办单位(即项目批准或申办者): Secondary sponsor:	* 国家: 中国<br>* Country: China<br>* 单位: 四川锦欣西囡妇女儿童医院<br>每格只填写一个单位(医院)<br>* Institution: Sichuan Jinxin Xinan Women's and Children's Hospital<br>Input only one institution<br>* 省(直辖市): 四川<br>* Province: Sichu<br>* 具体地址: 成都市<br>* Address: 66 Bis
* 经费或物资来源:	成都中医药大学杏林人才提升计划（YQRC2022001）
* Source(s) of funding:	Chengdu University of Traditional Chinese Medicine Xinglin Talent Enhancement Plan (YQRC2022001)
* 研究疾病:	反复种植失败（RIF）
* Target disease:	Rcurrent Implantation Failure (RIF)
* 研究类型: Study type:	观察性研究/Observational study
* 研究所处阶段: Study phase:	其它/N/A
* 研究目的:	本研究收集患者在冻融胚胎移植（FET）前一日2D/3D阴道超声检查的多维影像学特征，讨论<br>第一，MV-FLOW子宫内膜血管分布指数与/或内膜血管指数对FET结局的预测准确度?<br>第二，MV-FLOW子宫内膜血管分布指数与/或内膜血管指数能否用于分类反复种植失败患者与
* Objectives of Study:	

In this study, we collected multidimensional imaging features of patients' 2D/3D vaginal ultra
First, what is the predictive accuracy of the MV-FLOW endometrial vascular distribution inde
Second, can the MV-FLOW Endometrial Vessel Distribution Index and/or Endovascularity In
transplant success?

药物成份或治疗方案详述:

Description for medicine or protocol of treatment in detail:

* 纳入标准:

（1） 21-45岁之间，进行FET的女性；
（2） FI或RIF患者；
（3） 用单纯激素替代方案或GnRH-a降调节激素替代方案行内膜准备的患者；
（4） 自愿签署知情同意书。
注：同时满足以上5条标准即纳入本项研究。

* Inclusion criteria:

(1) Between 21-45 years of age, female undergoing FET;
(2) Diagnosed as FI or RIF;
(3) Patients undergoing endothelial preparation with hormone replacement alone or GnRH-a
(4) Voluntarily sign the informed consent form.
Note: Patients who met all 5 criteria were included in the study.

* 排除标准:

（1） 患有明确诊断的内、外科严重原发性或继发性疾病进行期或精神类疾病；
（2） 超声检查有未处理明确影响子宫形态的子宫畸形、压迫宫腔的子宫肌瘤或卵巢囊肿。
（3） 既往有任意部位的恶性肿瘤病史；
（4） 患者染色体异常（染色体多态性除外）。
注：凡符合以上任意1条者均予以排除。

* Exclusion criteria:

(1) A clearly diagnosed serious primary or secondary medical or surgical disease in progress
(2) Ultrasound with untreated uterine malformations that clearly affect uterine morphology, ut
(3) Previous history of malignant tumor of any site;
(4) Patients with chromosomal abnormalities (except for chromosomal polymorphisms).
Note: Anyone who meets any of the above criteria will be excluded.

* 研究实施时间:
Study execute time: 2024-07-15 - 2025-07-15

* 干预措施:
Interventions:

* 组别: 首次移植患者
* Group: FI patients
* 干预措施: 无
每格只填写一个干预措施
* Intervention: None
Please input only one Measure

* 组别: 反复种植失败患者
* Group: RIF patients
* 干预措施: 无
每格只填写一个干预措施
* Intervention: None
Please input only one Measure

* 样本总量:
Total sample size: 646

* 研究实施地点:
Countries of recruitment and research settings:

多中心研究完整填写所有分中心

* 国家:	中国
* 省(直辖市):	四川
* Country:	China
* Province:	Sichu
* 单位(医院):	四川锦欣西囡妇女儿童医院<br>每格只填写一个单位(医院)
* Institution(hospital):	Sichuan Jinxin Sinan Women's and Children's Hospital<br>Input only one institution

* 测量指标:
Outcomes:

每项指标分框填写，如有区分指标类型，请准确选择

* 指标中文名:	临床妊娠<br>每格只填写一个指标
* Outcome Name:	Clinical pregnancy<br>Input only one outcomes name
测量时间点:	
Measure time point of outcome:	

* 指标中文名:	生化妊娠<br>每格只填写一个指标
* Outcome Name:	Biochemical pregnancy<br>Input only one outcomes name
测量时间点:	
Measure time point of outcome:	

* 指标中文名:	胚胎种植<br>每格只填写一个指标
* Outcome Name:	Embryo implantation<br>Input only one outcomes name
测量时间点:	
Measure time point of outcome:	

* 采集人体标本:
Collecting sample(s) from participants:

填写标本名称即可，如不涉及可填'无'

* 标本中文名:	无<br>每格只填写一个，如"血液"、"唾液"等
* Sample Name:	None<br>Input only one,such as "Blood","Saliva" etc.
* 人体标本去向:<br>* Fate of sample:	其它/Others

征募研究对象情况:<br>Recruiting status:	尚未开始/Not yet recruiting
性别:<br>Gender:	女性/Female

* 随机方法（请说明由何人用什么方法产生随机序列:

	无
* Randomization Procedure (please state who generates the random number sequence and by what method):	None
研究对象是否签署知情同意书: Sign the informed consent:	○ 是/Yes ○ 否/No
隐蔽分组方法和过程:	如不涉及请填无/None
Process of allocation concealment	如不涉及请填无/None
盲法:	请说明施盲对象，如不涉及请填'无'
Blinding:	请说明施盲对象，如不涉及请填'无'
揭盲或破盲原则和方法:	如不涉及请填无/None
Rules of uncover or ceasing blinding:	如不涉及请填无/None
统计方法名称:	
Statistical method:	
是否公开试验完成后的统计结果: Calculated Results after the Study Completed public access:	不公开/Private
全球唯一识别码: UTN:	
* 是否共享原始数据: IPD sharing:	○ 是/Yes ○ 否/No
* 共享原始数据的方式(说明：请填入公开原始数据日期和方式，如采用网络平台，需填该网络平台名称和网址):	无

* The way of sharing IPD"(include metadata and protocol, If use web-based public database, please provide the url):	None
* 数据采集和管理（说明：数据采集和管理由两部分组成，一为病例记录表(Case Record Form, CRF)，二为电子采集和管理系统(Electronic Data Capture, EDC)，如ResMan即为一种基于互联网的EDC:	病历记录表。
* Data collection and Management (A standard data collection and management system include a CRF and an electronic data capture:	CRF
数据与安全监察委员会:<br>Data and Safety Monitoring Committee:	有/Yes
研究计划书或研究结果报告发表信息(杂志名称、期、卷、页，时间；或网址)::	
Publication information of the protocol/research results report(name of the journal, volume, issue, pages, time; or website):	
注册人:<br>Name of Registration:	刘瓅莹[248845]
注册时间:<br>Date of Registration:	2024-07-01 00:00:00
项目来源:<br>Date of Registration:	注册中心

提交submit　< 返回列表

提示：当您确认您的信息已经完整无误后，您可以点击"提交"按钮提请工作人员对该信息进行审核，否则您可以点击"保存"按钮保存信息但是暂不提请审核。

Tip: after you have completed the filling all the information in the registration form, you can select "Submit" button to apply for verification. Otherwise, select "Save" t
