# Supplementary figures and images for: Utilizing MV-FLOW™ and multidimensional ultrasound characteristics for prognosticating FET outcomes in RIF patients: Study Protocol for a cross-sectional study
Source: PLoS One. 2025 Feb 3;20(2):e0316028. doi: 10.1371/journal.pone.0316028 (PMC11790133; doi:10.1371/journal.pone.0316028)

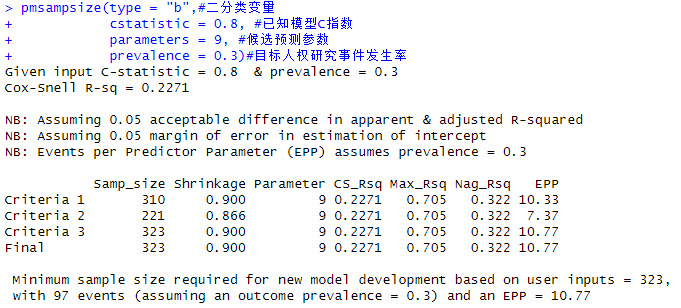

Supplement: S1 Fig — (PNG) [file pone.0316028.s003.png]
